# Comparative transcriptomics in human COPD reveals dysregulated genes uniquely expressed in ferrets

**DOI:** 10.1186/s12931-022-02198-0

**Published:** 2022-10-10

**Authors:** Shah S. Hussain, Yvonne J. K. Edwards, Emily Falk Libby, Denise Stanford, Stephen A. Byzek, Don D. Sin, Merry-Lynn McDonald, S. Vamsee Raju, Steven M. Rowe

**Affiliations:** 1 Department of Medicine, University of Alabama at Birmingham, MCLM 829 1918 University Blvd, Birmingham, AL 35294-0006 USA; 2 Department of Biochemistry & Molecular Genetics, University of Alabama at Birmingham, Birmingham, AL USA; 3 Department of Cell Developmental and Integrative Biology, School of Medicine, University of Alabama at Birmingham, Birmingham, AL USA; 4 Department of Pediatrics, School of Medicine, University of Alabama at Birmingham, Birmingham, AL USA; 5 Gregory Fleming James Cystic Fibrosis Research Center, University of Alabama at Birmingham, Birmingham, AL USA; 6 Centre for Heart Lung Innovation and Division of Respiratory Medicine, University of British Columbia, Vancouver, Canada

**Keywords:** COPD, Chronic bronchitis, Ferret model, Gene expression, Cigarette smoke

## Abstract

**Background:**

Chronic obstructive pulmonary disease (COPD) is a progressive lung disease with poor treatment options. However, most mouse models of COPD produce a primarily emphysematous disease not recapitulating clinically meaningful COPD features like chronic bronchitis.

**Methods:**

Wild-type ferrets (*Mustela putorius furo*) were divided randomly into two groups: whole body cigarette smoke exposure and air controls. Ferrets were exposed to smoke from 1R6F research cigarettes, twice daily for six months. RNA-sequencing was performed on RNA isolated from lung tissue. Comparative transcriptomics analyses of COPD in ferrets, mice, and humans were done to find the uniquely expressed genes. Further, Real-time PCR was performed to confirmed RNA-Seq data on multiple selected genes.

**Results:**

RNA-sequence analysis identified 420 differentially expressed genes (DEGs) that were associated with the development of COPD in ferrets. By comparative analysis, we identified 25 DEGs that are uniquely expressed in ferrets and humans, but not mice. Among DEGs, a number were related to mucociliary clearance (NEK-6, HAS1, and KL), while others have been correlated with abnormal lung function (IL-18), inflammation (TREM1, CTSB), or oxidative stress (SRX1, AHRR). Multiple cellular pathways were aberrantly altered in the COPD ferret model, including pathways associated with COPD pathogenesis in humans. Validation of these selected unique DEGs using real-time PCR demonstrated > absolute 2-fold changes in mRNA versus air controls, consistent with RNA-seq analysis.

**Conclusion:**

Cigarette smoke-induced COPD in ferrets modulates gene expression consistent with human COPD and suggests that the ferret model may be uniquely well suited for the study of aspects of the disease.

**Supplementary Information:**

The online version contains supplementary material available at 10.1186/s12931-022-02198-0.

## Background

Chronic obstructive pulmonary disease (COPD) is a heterogeneous disease diagnosed typically by irreversible airflow obstruction and persistent respiratory symptoms [1, 2]. COPD remains among the top five leading causes of death worldwide [3] and still lacks pharmacologic treatments that can mitigate COPD progression and persistent symptoms of the chronic bronchitis phenotype that include chronic cough and sputum production. Genetic studies can reveal novel mechanisms and suggest potential therapeutic strategies for investigation.

Cigarette smoking and chronic exposure to other inhaled pollutants are principal environmental risk factors driving COPD pathogenesis known to exert dysregulation of gene expression in the lung as well as extrapulmonary tissues [4–6]. However, transcriptomics findings in human studies cannot control for phenotypic variations that can be achieved using experimental systems, and animal studies have largely been limited to studies in mice [5, 6]. Mice are among the most utilized models to study COPD [7, 8], yet produce primarily very mild emphysematous disease and do not develop features pathognomonic for chronic bronchitis or mucus hypersecretion [9]^.^ To remedy this, we developed a cigarette smoke exposure model in ferrets that recapitulates features of emphysema as well as COPD-associated chronic bronchitis including airway remodeling, mucous metaplasia, muco-obstructive lung disease and periodic infectious exacerbations [10–13].

In the current report, we conducted transcriptome profiling of ferrets with COPD and compared findings to prior results from human COPD lungs [14] and a mouse model of COPD [6]. Overall, we sought to identify whether our ferret model of COPD captures unique genetic signatures in comparison to mouse models that can help improve understanding of the molecular pathogenesis of human COPD and promote the development of new and effective therapies.

## Materials and methods

### Cigarette smoke exposure in ferrets

Age and sex-matched ferrets (*Mustela putorious furo*) were procured from Marshall BioResources and exposed to room air or cigarette smoke generated by an automated cigarette smoke generator (TSE Systems, Chesterfield, MO). Smoke exposure consisted of 1 h of smoke from 1R6F research cigarettes, twice daily for at least 6 months. Animals were continuously monitored for particulate matter (200 ug/L) and CO levels (~ 1% to ~ 3%). All animal experiments were approved by the Institutional Biosafety Committee of the University of Alabama at Birmingham (IACUC 20232).

### RNA isolation

Following euthanasia, RNA was extracted from freshly frozen ferret lung samples using a RNeasy Mini Kit (Qiagen, Germantown, MD, USA) according to the manufacturer’s instructions. RNA integrity and concentration were examined prior to processing for RNA-seq.

### Next-generation sequencing on Illumina platforms

mRNA sequencing was performed on an Illumina NextSeq 500 System as described by the manufacturer (Illumina Inc., San Diego, CA, USA). Briefly, the quality of the total RNA was assessed using the Agilent 2100 Bioanalyzer. RNA with an RNA Integrity Number (RIN) of 7.0 or above was used for sequencing library preparation. We used the Agilent SureSelect Strand Specific mRNA library kit as per the manufacturer’s instructions (Agilent, Santa Clara, CA, USA). Library construction began with two rounds of polyA selection using oligo dT containing magnetic beads. The resulting mRNA was randomly fragmented with cations and heat, which was followed by first strand synthesis using random primers with inclusion of Actinomycin D (2.4 ng/µL final concentration). Second strand cDNA production was done with standard techniques and the ends of the resulting cDNA were made blunt, A-tailed and adaptors ligated for indexing to allow for multiplexing during sequencing. The cDNA libraries were quantitated using qPCR in a Roche LightCycler 480 with the Kapa Biosystems kit for Illumina library quantitation (Kapa Biosystems, Woburn, MA, USA) prior to cluster generation. Cluster generation was performed according to the manufacturer’s recommendations for onboard clustering (Illumina). Paired end 75 bp sequencing runs were completed to allow for better alignment of the sequences to the reference genome.

### RNA-seq analysis

**Fig. 1:**
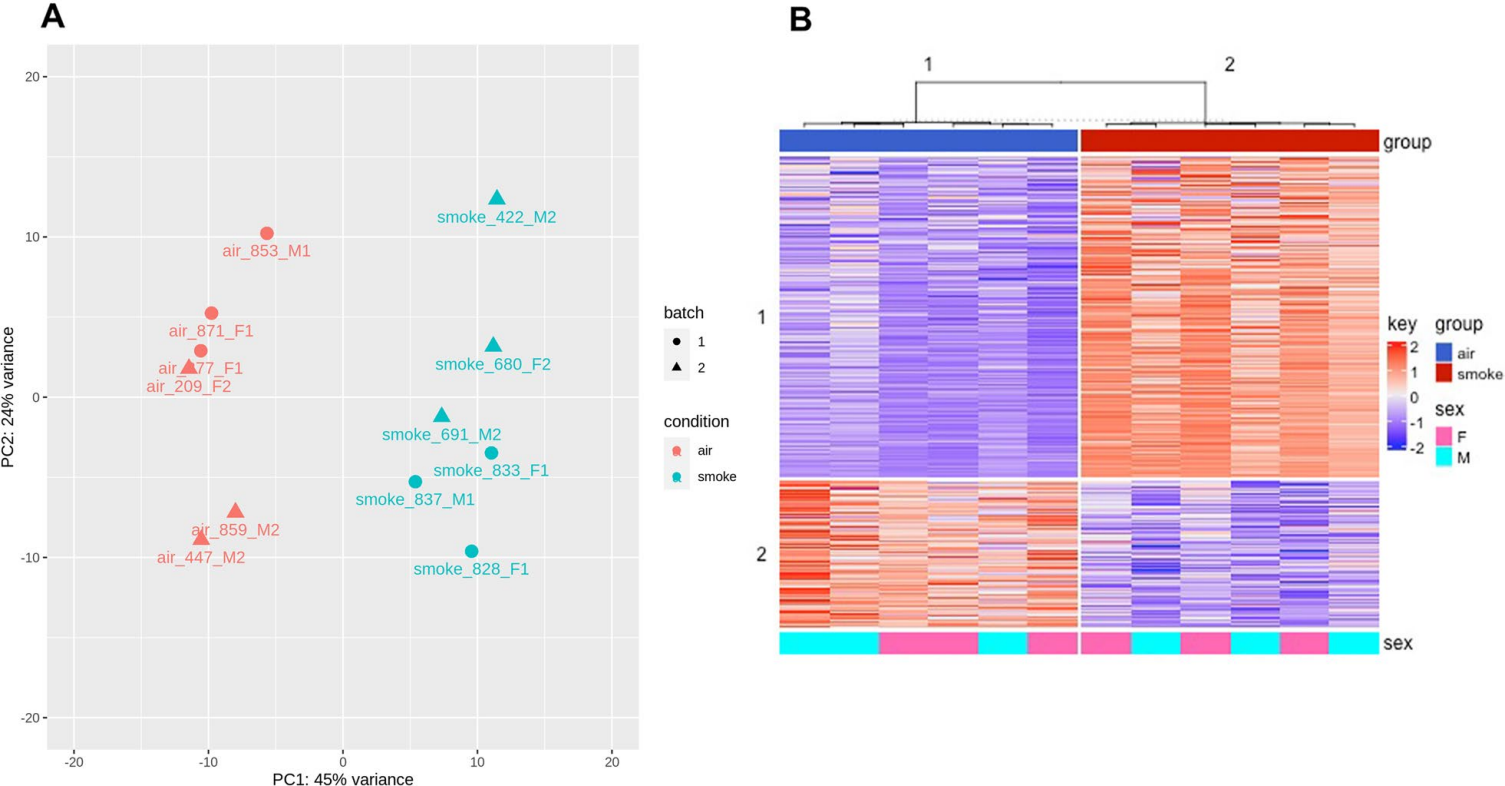
Differential expression pattern of ferret lungs following cigarette smoke exposure. Age and sex-matched ferrets (*Mustela putorious furo*) were exposed to cigarette smoke or air control for 6 months, after which lung tissues were isolated and RNA was extracted. Total RNA was isolated from lung homogenate and RNA-Seq analysis was performed. **A** Principal component analysis of the RNA seq data showing the distinct grouping of air control and smoke-exposed ferret lung (experimental) samples. **B** Hierarchical clustering of differentially expressed genes (DEGs). Studies were performed in two groups of 6 synchronous ferrets, each with 3 males and 3 females per group

RNA-seq data from 12 individual ferrets (six ferrets for air and smoke experimental conditions, respectively) were sequenced using an Illumina NextSeq 500 System (Additional file 1: Table S1). Paired end 75 bp reads were generated. Pre-alignment quality assessments of the raw fastq sequences were carried out using FastQC (version 0.11.7) (Andrews, 2010). The number of paired ends reads for the twelve samples range from 35 to 57 M (Additional file 2: Table S2, Additional file 3: Fig. S1). The raw fastq sequences were aligned to the *Mustela putorius furo* reference genome (GenBank assembly accession: GCA_000215625.1) [15]. The alignments were carried out using STAR (version 2.7.1a) [16]. (--outFilterType BySJout --outFilterMultimapNmax 20 --alignSJoverhangMin 8 --alignSJDBoverhangMin 1 --outFilterMismatchNmax 999 --outFilterMismatchNoverReadLmax 0.04 --alignIntronMin 20 -alignIntronMax 1000000 --alignMatesGapMax 1000000; as per ENCODE STAR parameter options for RNA-seq). Post-alignment quality assessments were carried out with RSeQC (version 2.6.3) [17]. Samtools (version 0.0.19) [18] and IGV (version 2.6.2) were used for indexing and viewing the alignments respectively. Gene expression was quantified as gene level counts using the htseq-count function (version 0.12.3) [19]. The Ensembl gene annotations for *Mustela putorius furo* (genebuild-last-updated 2016-05) was used [20]. The htseq-count default parameters were used except for the strand parameter which was set to reverse to consider the strandedness of library. DESeq2 filtered the low counts; genes for which there are less than 3 samples with normalized counts greater than or equal to 4 were filtered out. For each sample, the read counts were normalized using the Median Ratio Normalization method. Batch effects were evaluated using the ComBat function in sva (version 3.33.1) [21]; principal component analysis plots with the normalized gene expression data were used. Differentially expressed genes (DEGs) were identified using DESeq2 (version 1.28) [22]. DESeq2 was configured by adding batch and sex to the design to control for these factor whilst DESeq2 tests for association due to the condition [22]. Genes were differentially expressed if the absolute log_2_Fold change was > 1, and the adjusted p-value was < 0.1. The transformed normalized gene expression data with batch removal and adjustments for sex, using the limma function removeBatchEffect (version 3.44.0) (Ritchie et al. 2015), was used for downstream analyses (such as clustering analysis and heatmap generation (Fig. 1). The complex heatmap package (version 2.7.1.1015) [23] was used to generate heatmaps. The volcano plots were generated with an R Bioconductor package EnhancedVolcano (version 1.12.0) [24]. Circos plots were drawn with Circos (version 0.699) [25]. Interactivenn was used to generate Venn diagrams [26].

### Functional enrichment analysis

To assess which biological functions were altered upon smoke exposure, over-representation enrichment analysis was performed using the WEB-based GEne SeT AnaLysis Toolkit (Webgestalt) [27]. The functional database selected was gene ontology (biological process); the genome was selected as the reference set. A list of gene symbols was supplied (those genes that mapped between the ferret and human [20]; the genes supplied were DEGs. The organism of interest selected was Human. The hypergeometric test was used for over-representation evaluation for the lists of statistically altered DEGs. The Benjamini and Hochberg method was used to calculate the adjusted p-values (q). Other functional databases used were the gene ontology for molecular function and the biological pathway (KEGG). The detection of over-represented gene ontologies in the differentially expressed genes was also performed using the clusterProfiler package (version 4.0) [28, 29].

### Real-time PCR validation

For qRT-PCR analysis, RNA was isolated using miRNeasy Mini Kit as per manufacturer protocol (Qiagen, Cat No.: 217004, USA). 50 nanogram of RNA was used for further amplification by using TaqMan™ RNA-to-CT™ 1-Step Kit (Thermo Scientific). Analyses of mRNA levels were carried out using StepOnePlus Real-Time PCR System (Life Technologies, Carlsbad, CA, USA), TaqMan Universal PCR Mastermix (Applied Biosystems, Waltham, MA, USA) and gene-specific Prime PCR Probe Assays was designed and order from thermofisher (Assay ID: APEPYE2, APT2FKD, APU696A, APFVTYY, APGZMJW, APH6F4U, APKCAPR, APMF4AN, APNKXVK, APPRTFH and APRWKZF). GAPDH was used for reference housekeeping gene. Relative expressions were quantified with the 2^−ΔΔCT^ method [30].

### Data access

The experiments performed in this study are available in GEO under the accession number (GSE193749).

## Results

### Evaluation of differentially expressed genes in smoke-exposed ferret lungs

**Fig. 2:**
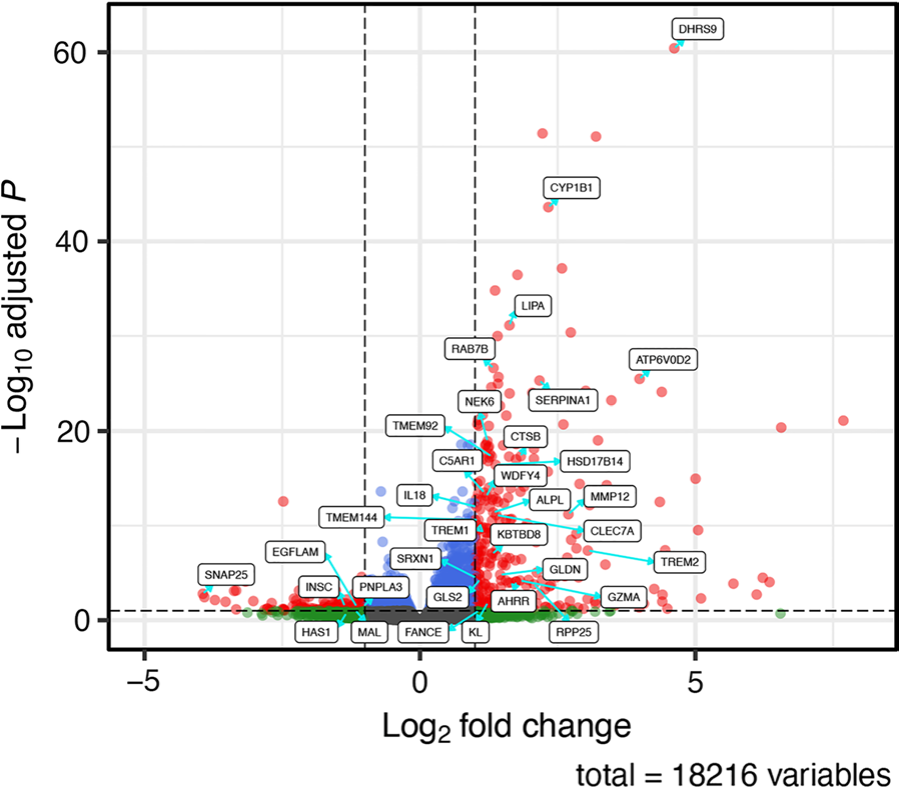
Volcano plot of differentially expressed genes (DEGs) in smoke-exposed ferrets. Differential expression volcano plot adjusted for batch and sex. Genes were differentially expressed if the absolute log_2_fold change > 1 and the adjusted p-value < 0.1; these are plotted as red dots. The known cigarette smoke exposure marker *CYP1B1* and other genes that have biological or scientific basis in smoking or COPD are indicated by text

The transcriptome profile of COPD ferrets has not been previously reported. To determine the gene expression signature of the ferret model, lung parenchyma was derived from cigarette smoke-exposed or air control ferrets and assessed using RNA-Seq. In the ferret transcriptomics data, the overall read mapping rate to the ferret reference genome was between ranged between 80 and 92% and did not significantly vary by smoking status. Principal component analysis of global transcriptomics demonstrated clear discrimination of smoke and control exposed ferrets (Fig. 1A)**.** Hierarchical clustering of DEGs, shown by heat map after adjustment of transcript levels by batch and sex (Fig. 1B), demonstrated distinct expression patterns by exposure condition, with pronounced alterations of the transcriptome in response to smoke, in smoke exposed ferrets. Overall, a total of 420 DEGs were associated with smoke exposure in COPD ferrets (Fig. 2, Additional file 1: Table S1) Cytochrome P450 family 1 subfamily B member 1 (CYP1B1) was highly upregulated in the model (5.1-fold increase, Benjamini–Hochberg adjusted p-value = 2.41E−44), in line with previous observations in humans with COPD [31] and rodent exposure models [32], and thus served as an important quality control.

### Gene ontology enrichment analysis

**Fig. 3:**
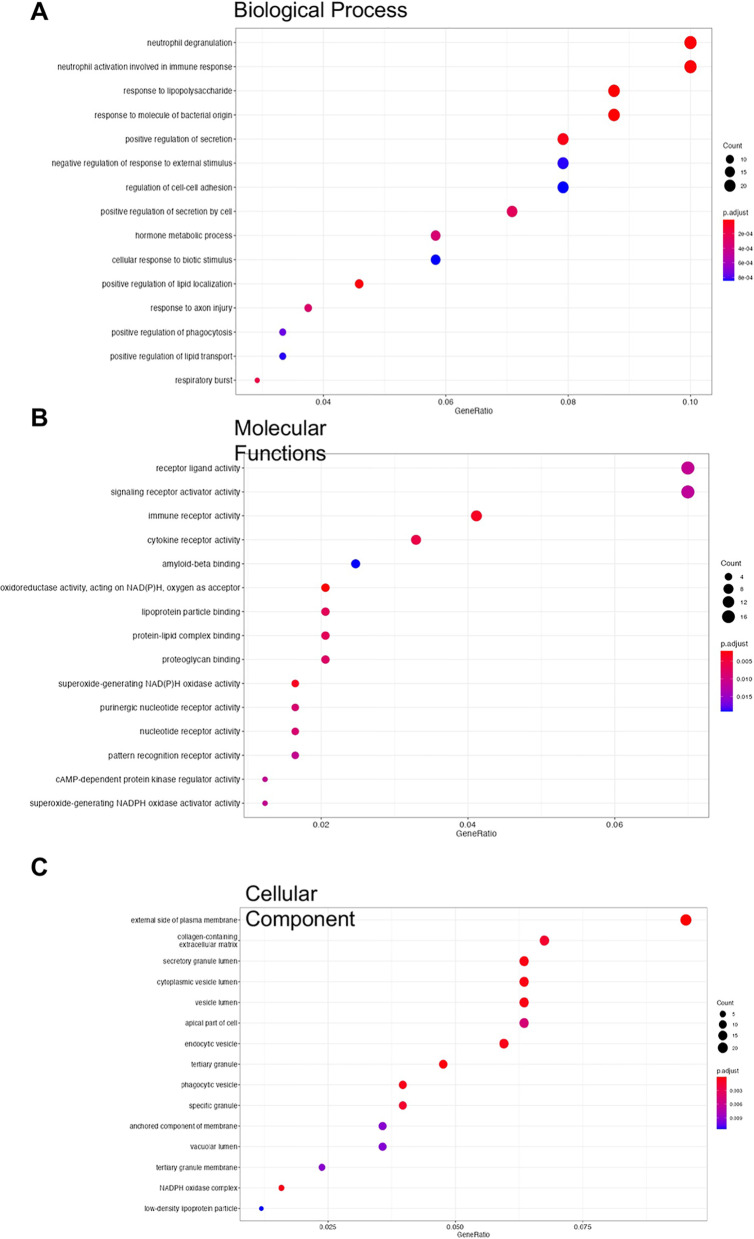
Gene ontology (GO) annotation plots for differentially expressed genes (DEGs) identified in smoke-exposed ferrets. Bubble plots showing GO terms affected for **A** biological Process, **B** molecular function, and **C** cellular component categories, by DEG count and FDR in smoke exposed ferrets as compared to controls

To support these pathway analyses, we further acquired an overview of Gene Ontology by evaluating the functional annotations of ferret DEGs based on GO terms. The top 15 associated functions in each component are presented in Fig. 3 and the complete annotation is provided in Additional file 4: Table S3. Disease relevant pathways that were altered in biological processes (GO: BP) prominently included neutrophil degranulation, neutrophil activation, and respiratory burst, whereas G protein-coupled receptor signaling pathway and cell adhesion were down regulated (Fig. 3A; Additional file 4: Table S3). Molecular functions (GO: MF) notable included receptor signaling pathways and oxidoreductase activity (Fig. 3B; Additional file 4: Table S3), whereas cellular component GO terms included extracellular matrix and NADPH oxidase (Fig. 3C; Additional file 4: Table S3).

### Functional and gene ontology (GO) enrichment analysis of differentially expressed genes in smoke-exposed ferret lungs

DEGs associated with cigarette smoke exposure identified from the analysis of ferret lung were next subjected to functional enrichment analysis. Top ten upregulated and downregulated KEGG pathways are shown in Table 1 in rank order by p-value, noting many were not statistically significant by FDR.

**Table 1:** KEGG pathway analysis showing up-regulated (A) and down-regulated (B) pathways

(A) Up-regulated genes—KEGG							
Gene set	Description	Size	Overlap	Expect	ER	p-value	FDR
hsa04145	Phagosome	152	10	1.91	5.23	1.89E−05	0.004
hsa04142	Lysosome	123	9	1.55	5.81	2.18E−05	0.004
hsa04610	Complement and coagulation cascades	79	5	0.99	5.03	0.003070148	0.250
hsa05144	Malaria	49	4	0.62	6.49	0.003233764	0.250
hsa04611	Platelet activation	123	6	1.55	3.88	0.004416005	0.250
hsa04380	Osteoclast differentiation	128	6	1.61	3.72	0.005361306	0.250
hsa05323	Rheumatoid arthritis	90	5	1.13	4.41	0.005373572	0.250
hsa04210	Apoptosis	136	6	1.71	3.51	0.007171521	0.266
hsa04640	Hematopoietic cell lineage	97	5	1.22	4.10	0.007356998	0.266
hsa04060	Cytokine-cytokine receptor interaction	294	9	3.70	2.43	0.011381961	0.347

(B) Down-regulated genes—KEGG							
Gene set	Description	Size	Overlap	Expect	ER	p-value	FDR
hsa04060	Cytokine-cytokine receptor interaction	294	8	1.30	6.16	3.08E−05	0.01
hsa04080	Neuroactive ligand-receptor interaction	277	6	1.22	4.90	0.001172172	0.19
hsa04930	Type II diabetes mellitus	46	2	0.20	9.84	0.017356504	1.00
hsa04672	Intestinal immune network for IgA production	49	2	0.22	9.24	0.01955958	1.00
hsa03320	PPAR signaling pathway	74	2	0.33	6.12	0.041960528	1.00
hsa04020	Calcium signaling pathway	183	3	0.81	3.71	0.046110736	1.00
hsa04062	Chemokine signaling pathway	189	3	0.84	3.59	0.049930188	1.00
hsa04350	TGF-beta signaling pathway	84	2	0.37	5.39	0.052704308	1.00
hsa04974	Protein digestion and absorption	90	2	0.40	5.03	0.059574237	1.00
hsa04750	Inflammatory mediator regulation of TRP channels	99	2	0.44	4.57	0.07042317	1.00

Top ten gene sets annotated are displayed

*ER* enrichment ratio; *FDR* False Discovery Rate

### Comparison of differentially expressed genes in smoke-exposed ferret lungs, mouse lungs, and human COPD

**Fig. 4:**
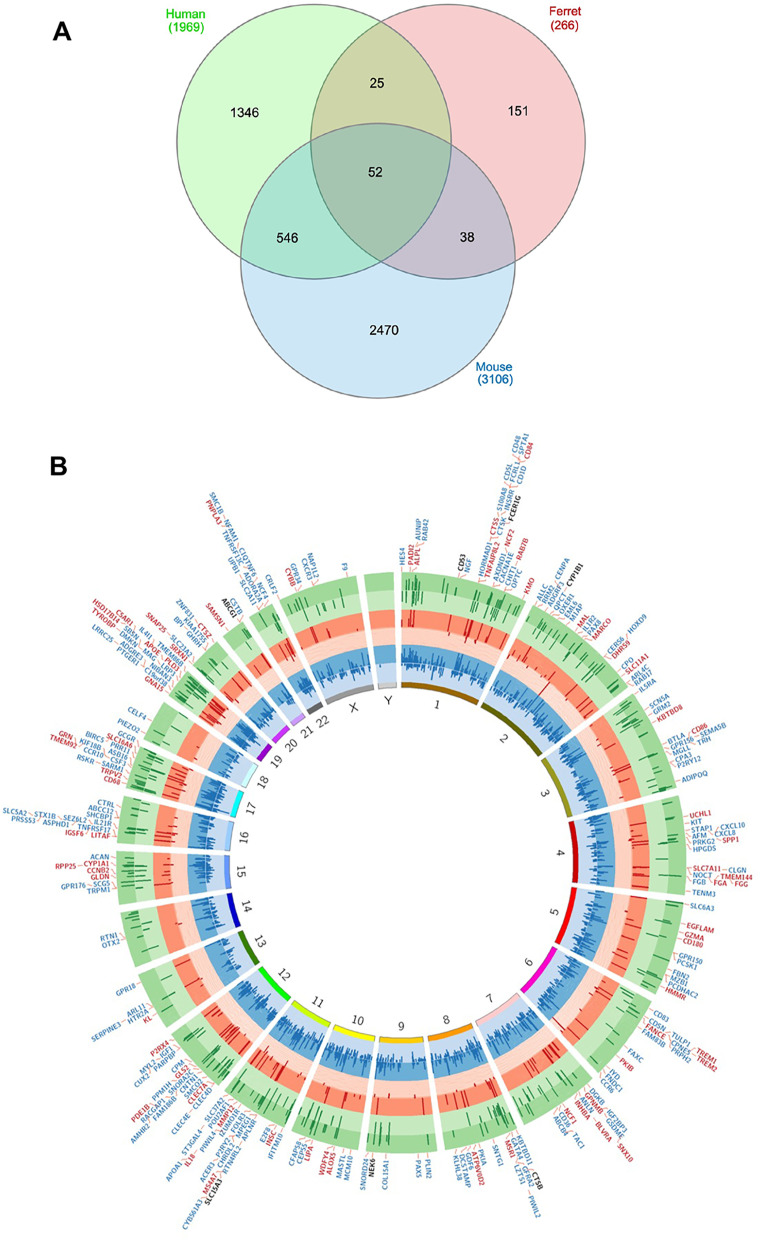
**A** Venn diagram depicting overlap between differentially expressed genes (DEGs) in smoke-exposed ferret, human COPD, and smoke-exposed murine lungs. The number of DEGs in common between smoke-exposed ferrets, human COPD [14], and smoke-exposed mice [6] are shown. **B** Circos plot of smoking-related genes overlapping between smoke-exposed ferret, human COPD, and smoke-exposed murine lungs. The gene chromosomal positions are based on the human genome and shown inside the circles. The first pair of circles (light blue and dark blue) represents the dysregulated genes from the long term (24 weeks) smoke-exposed mouse [6]. The upregulated genes are plotted in the dark blue circle pointing outward whilst the downregulated genes are plotted in the light blue circle pointing inward. The second pair of circles (light red and dark red) show the ferret genes dysregulated due to smoke exposure. The upregulated genes are in the dark red circle and the downregulated genes are in the light red circle. The third pair of circles (light green and dark green) show the “human smoking” gene signature [6, 14]. The upregulated genes are in the dark green circle, whilst the downregulated genes are in the light green circle. The length of the line is proportional to the −log_10_ FDR values for differential expression in human and for the −log_10_ FDR values in murine and for the −log_10_ p_adj_ for the ferret. Genes highlighted are statistically significant from the respective studies

A unique property of the COPD ferret model is that it exhibits classical pathophysiological features of airway disease, including goblet cell hyperplasia, mucus hyperexpression, and delayed mucus clearance, as well phenotypical features of chronic bronchitis including cough [10–12]. We next sought to understand whether COPD ferrets can provide insights into the molecular basis of human COPD that otherwise cannot be examined in COPD mouse models. We next sought to evaluate the extent of overlap between the genetic signatures of DEGs in ferrets as compared to human and/or mice. To do that, overlap was assessed between the COPD ferret gene expression signature and DEGs in datasets from Obeidat et al. evaluating mice that were smoke-exposed for 24 weeks duration [6] and Bosse et al. that evaluated humans with severely affected COPD [14], a population most relevant to the duration and intensity of chronic smoke exposure experienced by ferrets [12]. Of the 420 DEGs identified in COPD ferrets, 266 DEGs also had analyzable gene expression data generated in human [14] and mouse [6] models enabling comparative transcriptomic analyses between ferret, human, and mouse lungs. As depicted in Fig. 4A Venn diagram and detailed in the Circos plot in Fig. 4B that illustrates commonalities between the ferret, human, and mouse genomes, 52 (18.5%) of these 266 DEGs in ferrets were common to all three species, whereas 77 (28.9%) were also differentially expressed in humans, and 90 (33.8%) were differentially expressed in mice (Top 25 listed in Table 2; Complete list in Additional file 5: Table S4). Interestingly, 25 DEGs were differentially expressed in human and ferret, but not mice (Table 3); among these genes uniquely modeled in ferrets were those previously determined to be relevant to COPD pathogenesis, with upregulation observed for IL-18, KL, SRXN1, TREM1 and downregulation observed for HAS1 and PNPLA3 [33–36]. Of note, the change in KL and PLPLA3 expression in ferret were discordant with changes in humans found by Bosse et al. Among the 25 most statistically significant DEGs common in ferret and human, three other genes also showed discordant regulation between the two species: GZMA, SNAP25, and EGFLAM (Table 3). These genes are important in airway goblet cells, although studies of its expression and localization in ferrets have not been performed.

**Table 2:** The top 25 smoke related DEGs identified in the ferret, mouse and human lungs shown in order of statistically significance for the ferret study

FERRET	log_2_FC	p-value	P_adj_	MOUSE	log_2_FC	p-value	p_adj_	HUMAN	Wilcox p-value	Fold change
CD84	2.22	4.64E−56	3.81E−52	CD84	1.40	2.11E−13	1.01E−10	CD84	2.14E−09	1.40
CYP1B1	2.33	5.85E−48	2.41E−44	CYP1B1	0.89	9.33E−11	1.28E−08	CYP1B1	1.14E−19	4.53
ATP6V0D2	3.99	2.70E−29	3.42E−26	ATP6V0D2	1.04	1.11E−06	3.18E−05	ATP6V0D2	7.64E−16	5.89
PLD3	1.30	2.55E−28	2.62E−25	PLD3	1.23	8.27E−17	4.37E−13	PLD3	1.17E−07	1.32
CD180	1.63	1.44E−27	1.19E−24	CD180	0.39	4.42E−03	2.42E−02	CD180	1.66E−08	1.59
IGSF6	1.14	2.64E−25	1.89E−22	IGSF6	0.37	1.90E−03	1.24E−02	IGSF6	5.24E−14	1.96
TYROBP	1.57	3.37E−25	2.31E−22	TYROBP	0.77	3.38E−08	1.85E−06	TYROBP	7.77E−12	1.37
P2RX4	1.04	3.09E−24	1.89E−21	P2RX4	0.30	3.68E−05	5.37E−04	P2RX4	3.39E−10	1.36
INHBA	2.60	3.39E−24	1.99E−21	INHBA	1.02	8.99E−09	6.30E−07	INHBA	2.90E−10	1.66
MS4A7	1.25	5.20E−24	2.85E−21	MS4A7	1.59	4.51E−13	1.99E−10	MS4A7	2.74E−10	1.38
UCHL1	3.23	1.88E−22	9.68E−20	UCHL1	0.51	6.05E−07	1.94E−05	UCHL1	6.11E−11	2.08
NEK6	1.22	2.37E−22	1.18E−19	NEK6	0.71	5.95E−08	2.94E−06	NEK6	1.27E−16	2.05
CD68	1.75	4.96E−22	2.40E−19	CD68	1.75	4.27E−14	2.78E−11	CD68	7.47E−11	1.84
MSR1	2.07	1.95E−21	7.46E−19	MSR1	1.20	2.13E−04	2.20E−03	MSR1	4.05E−11	1.71
SLC15A3	1.27	7.11E−21	2.66E−18	SLC15A3	0.92	7.71E−13	2.81E−10	SLC15A3	2.20E−12	1.55
GRN	1.09	7.73E−21	2.83E−18	GRN	0.62	2.10E−12	5.69E−10	GRN	4.90E−10	1.39
CTSB	1.83	1.32E−20	4.64E−18	CTSB	0.61	1.22E−11	2.47E−09	CTSB	1.56E−11	1.55
SLC11A1	1.74	3.12E−20	9.88E−18	SLC11A1	1.13	2.93E−10	3.53E−08	SLC11A1	1.46E−10	1.79
CD53	1.13	3.25E−20	1.01E−17	CD53	0.58	1.50E−06	3.93E−05	CD53	2.08E−09	1.27
CTSS	1.24	4.99E−20	1.52E−17	CTSS	0.60	1.82E−07	7.35E−06	CTSS	2.24E−08	1.30
SLC7A11	3.39	2.09E−17	5.37E−15	SLC7A11	1.87	4.24E−12	1.09E−09	SLC7A11	1.13E−09	1.71
LITAF	1.25	3.89E−17	9.69E−15	LITAF	0.43	1.49E−05	2.61E−04	LITAF	7.65E−10	1.26
BLVRA	1.03	6.85E−16	1.45E−13	BLVRA	0.43	8.24E−08	3.82E−06	BLVRA	8.95E−08	1.26
CD86	1.11	9.72E−16	1.97E−13	CD86	1.18	1.57E−09	1.37E−07	CD86	6.29E−14	1.76
CTSZ	1.18	1.15E−15	2.30E−13	CTSZ	1.09	2.76E−14	2.08E−11	CTSZ	5.38E−08	1.32

The statistical metrics reported for the mouse and human are computed from the mouse 24 week smoke exposed study [6] and the human smoking study [14] respectively. The log_2_FC is log_2_ fold change; the p_adj_ is adjusted p-value

**Table 3:** 25 Smoke-related DEGs identified in the ferret and human lungs shown in order of statistically significance for the ferret study

FERRET	log_2_FC	p-value	p_adj_	Human	Wilcox p-value	Fold change
DHRS9	4.62	2.31E−65	3.80E−61	DHRS9	3.38E−08	1.70
LIPA	1.62	3.60E−35	7.41E−32	LIPA	1.40E−12	2.16
RAB7B	1.33	1.63E−30	2.43E−27	RAB7B	8.24E−09	1.52
TMEM92	1.30	1.38E−20	4.73E−18	TMEM92	3.28E−10	1.48
HSD17B14	1.37	1.28E−19	3.84E−17	HSD17B14	1.51E−12	1.69
C5AR1	1.17	1.42E−16	3.25E−14	C5AR1	4.37E−12	1.42
WDFY4	1.21	3.60E−16	7.90E−14	WDFY4	1.44E−07	1.29
IL18	1.03	5.78E−15	1.03E−12	IL18	2.26E−10	1.42
ALPL	1.33	2.34E−14	4.04E−12	ALPL	2.71E−09	1.39
CLEC7A	1.39	4.59E−14	7.62E−12	CLEC7A	2.62E−12	1.35
TMEM144	1.06	1.63E−13	2.50E−11	TMEM144	4.05E−11	1.38
TREM1	1.12	1.38E−12	1.84E−10	TREM1	2.24E−08	1.41
KBTBD8	1.31	1.14E−09	9.94E−08	KBTBD8	2.97E−09	1.61
GLDN	1.46	2.83E−07	1.57E−05	GLDN	2.28E−10	1.44
SRXN1	1.02	6.73E−07	3.38E−05	SRXN1	5.16E−08	1.36
GLS2	1.09	1.04E−06	4.97E−05	GLS2	2.46E−12	1.54
**GZMA**	1.81	1.36E−06	6.24E−05	GZMA	5.53E−08	0.65
RPP25	2.07	3.87E−06	1.61E−04	RPP25	4.64E−09	1.33
**SNAP25**	− 3.94	5.62E−05	1.63E−03	SNAP25	2.56E−09	2.10
**KL**	1.23	7.98E−04	1.43E−02	KL	1.80E−08	0.62
INSC	− 1.36	2.44E−03	3.37E−02	INSC	1.75E−07	0.72
**PNPLA3**	− 1.03	2.52E−03	3.46E−02	PNPLA3	7.50E−13	2.34
**EGFLAM**	− 1.15	2.75E−03	3.69E−02	EGFLAM	1.01E−06	1.52
MAL	− 1.17	9.75E−03	9.24E−02	MAL	2.72E−07	0.73
FANCE	1.07	1.07E−02	9.85E−02	FANCE	8.44E−11	1.30

The statistical metrics reported for the human are computed from the human smoking study [14]. The log_2_FC is log_2_ fold change; the p_adj_ is adjusted p-value. Genes listed in bold are discordant between ferret and human

### RT-PCR validation analysis of key genes in COPD ferret model

**Fig. 5:**
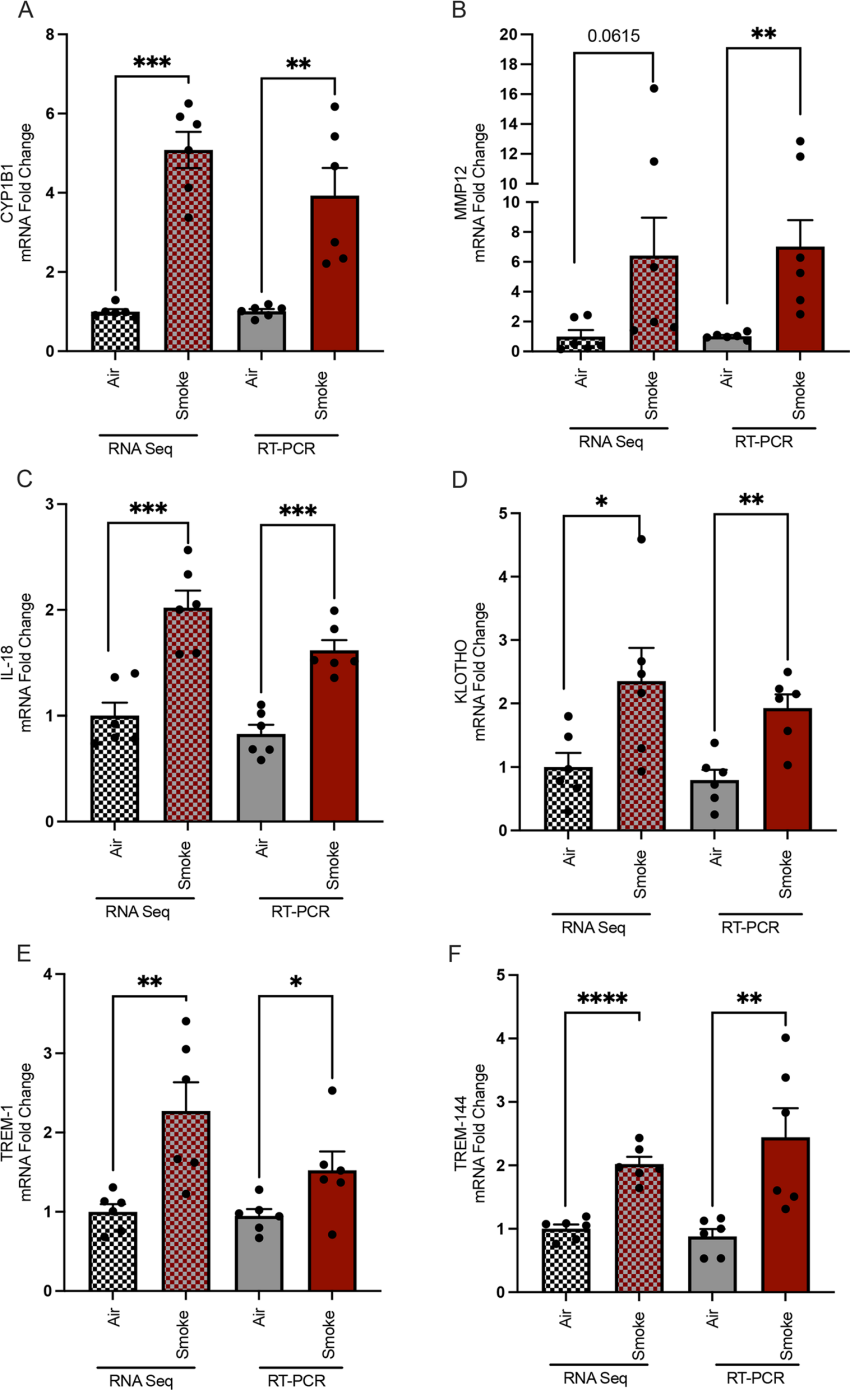

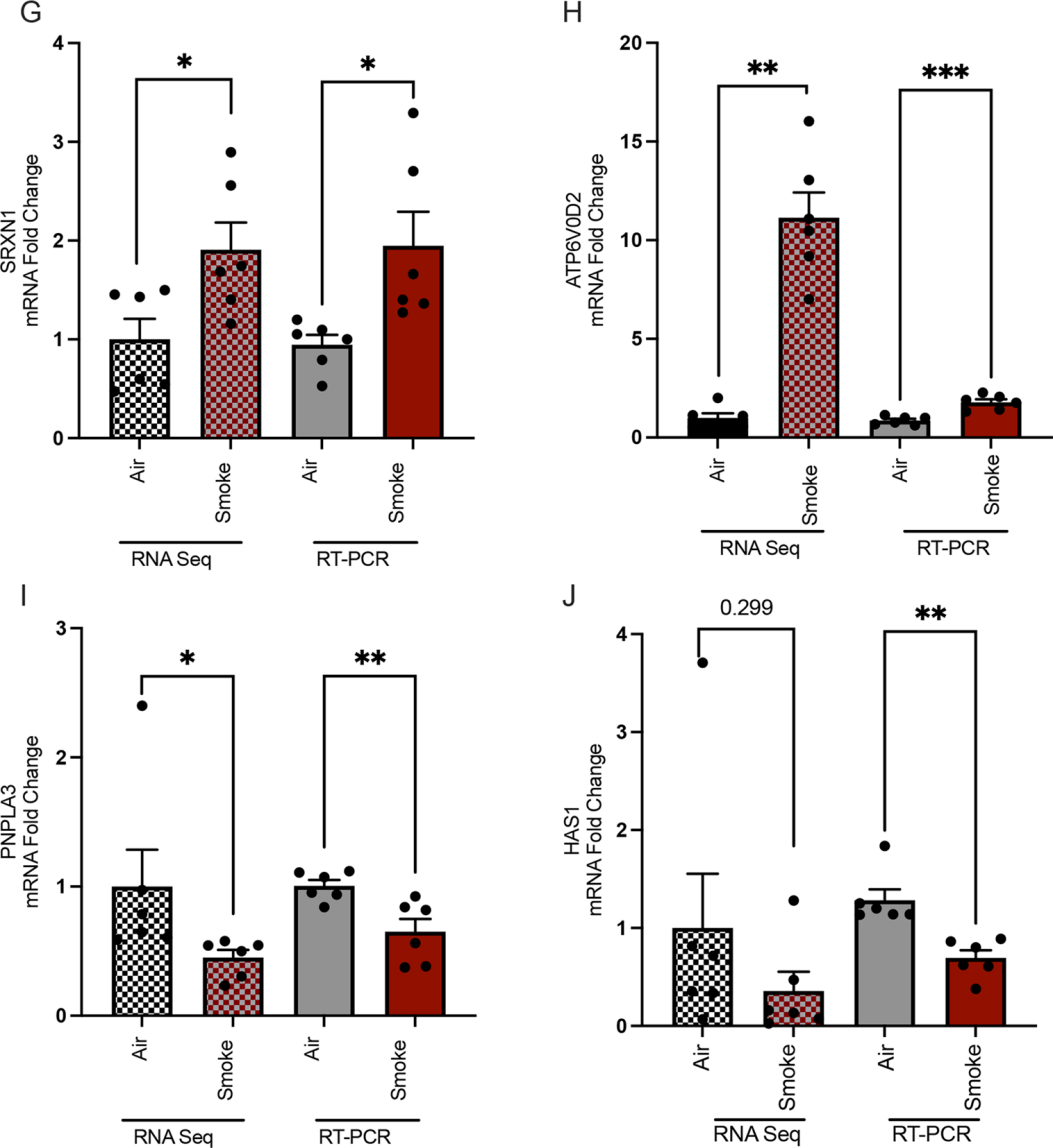
RT-PCR validation of differentially expressed genes (DEGs) unique to smoke-exposed ferret and human COPD lungs as compared to raw data from RNA-Seq. RT-PCR (right) and RNA-Seq (left) measurements for **A** CYP1B1; **B** MMP12; **C** IL-18; **D** KL; **E** TREM1; **F** TREM144; **G** SRXN1; **H** ATP6V0D2; **I** PNPLA3; and **J** HAS1 in smoke-exposed and air control ferret lungs. Mean ± SEM. RT PCR N = 6/condition, 3 males and 3 females per group, comparison by Mann–Whitney

To validate RNA-Seq results, we conducted RT-PCR using ferret tissues for selected DEGs (Fig. 5) that were uniquely modeled in ferrets, in addition to CYP1B1 as an exposure control, MMP12 as an important gene associated with COPD progression, and HAS1 as a disease of interest related to mucosal defense. Quantification of mRNA expression in smoke-exposed vs. air control ferrets by RT-PCR demonstrated expected changes with smoke exposure (Fig. 5), generally consistent with the RNA-seq analysis and providing validation for the utility of COPD ferrets in modeling a set of genes that can uniquely inform on the pathogenesis of COPD.

## Discussion

Various studies using cigarette smoke induced animal models have demonstrated genes associated with smoking by transcriptomic analysis [4, 5]. However, comparative studies using smoke exposed mouse models of COPD have limitations, since mice do not exhibit all features of the complex human COPD sub-phenotypes. In this study, we assessed the transcriptome profile of lung parenchyma in a ferret model of cigarette smoke-induced COPD. Gene expression profiling has not been previously reported in COPD ferrets, which uniquely exhibit features of airway remodeling in comparison to rodent models of cigarette smoke exposure, while also exhibiting emphysema [10–13]. A total of 420 DEGs associated with smoke exposure were identified, many of which have been observed in mice, humans, or both. Furthermore, building upon a recent study by Obeidat et al. who compared the expression profiles of smoke exposed (both short term and long term) murine model with COPD human lungs [6], we identified multiple additional DEGs and associated pathways bearing pathophysiological relevance to COPD that were uniquely expressed in ferrets and humans, but not mice. To our knowledge, this is currently the only RNA-seq based transcriptomics analysis in the COPD ferret model, and due to the unique features of ferrets, provides opportunities for detection of novel pathways with human relevance.

A major goal of the analysis was to identify gene targets that might be uniquely modeled in ferrets, given the human-like phenotype it exhibits. Our analysis revealed 25 aberrantly regulated genes that were exclusive to humans and the ferret COPD model, but not mice. These included several DEGs that have previously been studied for their role in COPD and that may be implicated in mucus-related aspects of the disease. IL-18 has been correlated with pulmonary function in COPD patients [37] and, in an IL-18 transgenic mouse model, was identified as key driver regulating downstream responses including mucus metaplasia, airway fibrosis, and vascular remodeling [38]. KL-deficient mice have been observed to exhibit delayed mucociliary clearance [34], and downregulation of KL protein KLOTHO has been widely observed in COPD with cigarette exposure [39, 40]. That we observed increased expression of KL may indicate that smoke exposed ferrets are responding to delayed mucociliary clearance, as recently shown [34], The discordant expression of KL in ferrets compared to humans could be due to the possibility that higher expression of KL acts as a compensatory response to oxidative stress due to smoking, a finding reported by other studies [41], including in human smokers without COPD [42]. Other DEGs common to humans and ferret of interest included the antioxidant enzyme sulfiredoxin-1 (SRX1), which was shown to be upregulated in the lungs of nuclear factor erythroid-2-related factor 2 (Nrf^+/+^) mice upon acute exposure to cigarette smoke, conferring a protective effect against oxidative stress, although was decreased in lung tissue from patients with advanced COPD [43]. This may have been prominent since ferrets exhibit a mild form of disease, given their age and relative duration of cigarette smoke exposure. Upregulation of triggering receptor expressed on myeloid cells 1 (TREM1) has been observed in cigarette-smoke mice, exacerbating pyroptosis-mediated lung injury and inflammation [44]. These pathways could represent opportunities for further investigation in COPD pathogenesis using the ferret model.

Prior investigations evaluating gene expression profiles of smoked-exposed mouse [6] and human COPD [14] lungs enabled cross-species transcriptomics analysis for 266 of the 420 total DEGs detected in the COPD ferret model. There was substantial overlap in DEGs detected across all three species, with involvement seen in genes with widely studied roles in COPD such as CYP1B1 [31], matrix metalloproteinase 12 (MMP12) [45], and aryl hydrocarbon receptor repressor (AHRR) [46], underscoring the validity of the smoke exposure model in ferrets for evaluation of the lung transcriptome. Common DEGs between ferret, human, and mouse also reinforced the contribution of additional genes to COPD pathogenesis. Among other overlapping DEGs, TREM2 has been observed to promote macrophage survival in the lung [47], nimA-related protein kinase 6 (NEK6) has been implicated in COPD-associated ciliopathies [48], and cathepsin B (CTSB) induced emphysema and secretory cell hyperplasia upon intratracheal administration to hamster lungs [49]. Additional genes of interest that were substantially upregulated and have direct relevance to COPD and/or cigarette smoke exposure included SERPINA1 (2.17 log_2_ fold change (log_2_FC); p_adj_ = 5.13E−26), ATP6V0D2 (3.99 log_2_FC; p_adj_ = 3.42E−26), MMP-12 (2.69 log_2_FC ; p_adj_ = 6.20E−12), AHRR (1.74 log_2_FC; p_adj_ = 8.32E−05), and CTSB (1.83 log_2_FC; p_adj_ = 4.64E−18), among others [4, 50].

Downregulation of hyaluronic acid synthase 1 (HAS1) has been associated with abnormally low production of the high molecular weight form of HA (HMW-HA) in COPD [51]. While RNA-Seq revealed a trend, RT-PCR, conducted because of prior interest in this area, indicated downregulation of HAS1. Reduced HAS1, and thus lower HMW-HA, could also lead to abnormal HMW/LMW HA ratios, which would alter mucus clearance and also induce inflammation [51].

Multiple cellular pathways were aberrantly altered in the COPD ferret model, including pathways that are also associated with COPD pathogenesis in humans. Of the pathways were significant changes were observed (Table 1 and Additional file 4: Table S3), those that have been reported to be involved with COPD pathogenesis included activation of mitogen-activated protein kinase (MAPK), phosphatidylinositol 3 kinase/serine threonine protein kinase (PI3K-AKT), phagosome, cytokine-cytokine receptor interaction, extracellular matrix (ECM)-receptor interaction, chemokines, apoptotic signaling pathways, inhibition of transforming growth factor beta (TGF-ß), peroxisome proliferator-activated receptors (PPAR), and inflammatory mediator regulation of transient receptor potential (TRP) channel pathways [52].

Our study, while unique in the analysis of ferrets, is not without limitations. Ferrets are an outbred species, and thus genetic heterogeneity may have reduced the ability to detect some important DEGs, especially given the sample size encountered. We focused our analysis on parenchyma tissue, noting this was most similar to the specimens collected in humans and other species for which we compared, and this samples also include the small airways, where airway disease is prominent [12, 53]; nevertheless, the analysis of airway epithelia could be informative in the future, especially given pathological changes in the large airways of ferrets upon smoking [10–12]. Since ferrets are sexually dimorphic, it is not surprising that there were important differences by ferret sex, although this was controlled for in the differential gene analysis and included where possible in comparison to other data sets. Importantly, analysis of RNA has evolved throughout the study, providing a limitation in the inter-species comparisons. The RNA-Seq analysis and RT-PCR validation in this study was based on mRNA expression; validation by protein analysis will be useful in the future, noting some protein reagents in ferrets need further characterization. To assure specificity of our comparisons, we required at least one-fold change, in addition to statistical significance for the DEG to be included in the ferret study, which differed from those included in mice-human comparators, In a minority of cases for genes uniquely modeled in ferrets, the directional change in differential expression in ferrets and humans were discordant. Although this can be explained for KL due to the potential for evolving changes over time, further research will be needed for other genes where this was the case, such as GZMA, SNAP25, KL, PNPLA3 and EGFLAM.

## Conclusion

In summary, our analysis provides a large RNA-seq based transcriptome analysis set in COPD and involves a ferret model that uniquely exhibits features of chronic bronchitis. Our data revealed 420 DEGs, including multiple genes established as abnormal among COPD patients, such as pathways involving neutrophil activation, immune regulation, and oxidative metabolism. We also identified 25 DEGs that are uniquely expressed in ferrets and humans, but not mice, some of which implicate abnormal mucus clearance, a finding uniquely modeled by ferrets. Selected genes were validated with real time PCR, and these were concordant with the RNA-Seq expression. Together, the transcriptomic profile and comparison with other available models of COPD identifies a unique set of genes that could be relevant to disease pathogenesis. This high-resolution view of the lung transcriptome associated with COPD may ultimately provide a valuable resource for the research community and warrants further mechanistic investigations.

## Supplementary Information

**Additional file 1: Table S1.** RNA-seq data for 12 samples (six biological replicates each for air and smoke experimental conditions) sequenced using an Illumina NextSeq 500 System. [GEO Accession Number GSE193749].

**Additional file 2: Fig. S1.** The proportion of paired ends reads for the ferret lung samples, by individual ferrets.

**Additional file 3: Table S2.** The number of paired ends reads for the ferret lung samples.

**Additional file 4: Table S3.** Complete annotation GO TERMS in smoke exposed ferrets compared to controls.

**Additional file 5: Table S4.** Complete list (52) of DEGs in common between ferret, mice, and human transcriptome.

## Data Availability

The datasets analyzed during the current study are available from the corresponding author on reasonable request.

## Abbreviations

COPD: Chronic obstructive pulmonary disease
qPCR: Quantitative polymerase chain reaction
DEGs: Differentially expressed genes
KEGG: Kyoto Encyclopedia of Genes and Genomes
CYP1B1: Cytochrome P450 family 1 subfamily B member 1
GO: Gene Ontology
BP: Biological process
MF: Molecular functions
KL: KLOTHO
SRX1: Sulfiredoxin-1
TREM1: Triggering receptor expressed on myeloid cells 1
Nrf: Nuclear factor erythroid-2-related factor 2
MMP12: Matrix metalloproteinase 12
AHRR: Aryl hydrocarbon receptor repressor
NEK6: NimA-related protein kinase 6
HAS1: Hyaluronic acid synthase 1
HMW-HA: High molecular weight form of HA
MAPK: Mitogen-activated protein kinase
PI3K-AKT: Phosphatidylinositol 3 kinase/serine-threonine protein kinase
ECM: Extracellular matrix
TGF-ß: Transforming growth factor beta
PPAR: Peroxisome proliferator-activated receptors
TRP: Transient receptor potential

## References

[1] Rabe KF, Watz H (2017). Chronic obstructive pulmonary disease. Lancet.

[2] Chand HS, Vazquez-Guillamet R, Royer C, Rudolph K, Mishra N, Singh SP, Hussain SS, Barrett E, Callen S, Byrareddy SN (2018). Cigarette smoke and HIV synergistically affect lung pathology in cynomolgus macaques. J Clin Invest.

[3] Chronic obstructive pulmonary disease (COPD). World Health Organization. https://www.who.int/news-room/fact-sheets/detail/chronic-obstructive-pulmonary-disease-(copd). Accessed 29 Oct 2021.

[4] Morissette MC, Lamontagne M, Berube JC, Gaschler G, Williams A, Yauk C, Couture C, Laviolette M, Hogg JC, Timens W (2014). Impact of cigarette smoke on the human and mouse lungs: a gene-expression comparison study. PLoS ONE.

[5] Yun JH, Morrow J, Owen CA, Qiu W, Glass K, Lao T, Jiang Z, Perrella MA, Silverman EK, Zhou X, Hersh CP (2017). Transcriptomic analysis of lung tissue from cigarette smoke-induced emphysema murine models and human chronic obstructive pulmonary disease show shared and distinct pathways. Am J Respir Cell Mol Biol.

[6] Obeidat M, Dvorkin-Gheva A, Li X, Bosse Y, Brandsma CA, Nickle DC, Hansbro PM, Faner R, Agusti A, Pare PD (2018). The overlap of lung tissue transcriptome of smoke exposed mice with human smoking and COPD. Sci Rep.

[7] Stevenson CS, Birrell MA (2011). Moving towards a new generation of animal models for asthma and COPD with improved clinical relevance. Pharmacol Ther.

[8] Vlahos R, Bozinovski S (2014). Recent advances in pre-clinical mouse models of COPD. Clin Sci (Lond).

[9] Wright JL, Cosio M, Churg A (2008). Animal models of chronic obstructive pulmonary disease. Am J Physiol Lung Cell Mol Physiol.

[10] Stanford D, Kim H, Bodduluri S, LaFontaine J, Byzek SA, Schoeb TR, Harris ES, Nath HP, Bhatt SP, Raju SV, Rowe SM (2020). Airway remodeling in ferrets with cigarette smoke induced COPD using microCT imaging. Am J Physiol Lung Cell Mol Physiol.

[11] Lin VY, Kaza N, Birket SE, Kim H, Edwards LJ, LaFontaine J, Liu L, Mazur M, Byzek SA, Hanes J (2020). Excess mucus viscosity and airway dehydration impact COPD airway clearance. Eur Respir J.

[12] Raju SV, Kim H, Byzek SA, Tang LP, Trombley JE, Jackson P, Rasmussen L, Wells JM, Libby EF, Dohm E (2016). A ferret model of COPD-related chronic bronchitis. JCI Insight.

[13] Basil MC, Cardenas-Diaz FL, Kathiriya JJ, Morley MP, Carl J, Brumwell AN, Katzen J, Slovik KJ, Babu A, Zhou S (2022). Human distal airways contain a multipotent secretory cell that can regenerate alveoli. Nature.

[14] Bosse Y, Postma DS, Sin DD, Lamontagne M, Couture C, Gaudreault N, Joubert P, Wong V, Elliott M, van den Berge M (2012). Molecular signature of smoking in human lung tissues. Cancer Res.

[15] Peng X, Alfoldi J, Gori K, Eisfeld AJ, Tyler SR, Tisoncik-Go J, Brawand D, Law GL, Skunca N, Hatta M (2014). The draft genome sequence of the ferret (*Mustela putorius* furo) facilitates study of human respiratory disease. Nat Biotechnol.

[16] Dobin A, Davis CA, Schlesinger F, Drenkow J, Zaleski C, Jha S, Batut P, Chaisson M, Gingeras TR (2013). STAR: ultrafast universal RNA-seq aligner. Bioinformatics.

[17] Wang L, Wang S, Li W (2012). RSeQC: quality control of RNA-seq experiments. Bioinformatics.

[18] Li H, Handsaker B, Wysoker A, Fennell T, Ruan J, Homer N, Marth G, Abecasis G, Durbin R (2009). Genome Project Data Processing S: the sequence alignment/Map format and SAMtools. Bioinformatics.

[19] Anders S, Pyl PT, Huber W (2015). HTSeq–a Python framework to work with high-throughput sequencing data. Bioinformatics.

[20] Yates AD, Achuthan P, Akanni W, Allen J, Allen J, Alvarez-Jarreta J, Amode MR, Armean IM, Azov AG, Bennett R (2020). Ensembl 2020. Nucleic Acids Res.

[21] Leek JT, Johnson WE, Parker HS, Jaffe AE, Storey JD (2012). The sva package for removing batch effects and other unwanted variation in high-throughput experiments. Bioinformatics.

[22] Love MI, Huber W, Anders S (2014). Moderated estimation of fold change and dispersion for RNA-seq data with DESeq2. Genome Biol.

[23] Gu Z, Eils R, Schlesner M (2016). Complex heatmaps reveal patterns and correlations in multidimensional genomic data. Bioinformatics.

[24] Blighe KRS, Lewis M, EnhancedVolcano: publication-ready volcano plots with enhanced colouring and labeling. https://github.com/kevinblighe/EnhancedVolcano. 2018.

[25] Krzywinski M, Schein J, Birol I, Connors J, Gascoyne R, Horsman D, Jones SJ, Marra MA (2009). Circos: an information aesthetic for comparative genomics. Genome Res.

[26] Heberle H, Meirelles GV, da Silva FR, Telles GP, Minghim R (2015). InteractiVenn: a web-based tool for the analysis of sets through Venn diagrams. BMC Bioinformatics.

[27] Wang J, Vasaikar S, Shi Z, Greer M, Zhang B (2017). WebGestalt 2017: a more comprehensive, powerful, flexible and interactive gene set enrichment analysis toolkit. Nucleic Acids Res.

[28] Yu G, Wang LG, Han Y, He QY (2012). clusterProfiler: an R package for comparing biological themes among gene clusters. OMICS.

[29] Wu T, Hu E, Xu S, Chen M, Guo P, Dai Z, Feng T, Zhou L, Tang W, Zhan L (2021). clusterProfiler 4.0: a universal enrichment tool for interpreting omics data. Innovation (N Y).

[30] Livak KJ, Schmittgen TD (2001). Analysis of relative gene expression data using real-time quantitative PCR and the 2(-Delta Delta C(T)) Method. Methods.

[31] Yang D, Yan Y, Hu F, Wang T (2020). CYP1B1, VEGFA, BCL2, and CDKN1A affect the development of chronic obstructive pulmonary disease. Int J Chron Obstruct Pulmon Dis.

[32] Kim YS, Kokturk N, Kim JY, Lee SW, Lim J, Choi SJ, Oh W, Oh YM (2016). Gene profiles in a smoke-induced COPD mouse lung model following treatment with mesenchymal stem cells. Mol Cells.

[33] Nakanishi K, Nishida M, Taneike M, Yamamoto R, Moriyama T, Yamauchi-Takihara K (2021). Serum Klotho levels contribute to the prevention of disease progression. Int J Gen Med.

[34] Garth J, Easter M, Skylar Harris E, Sailland J, Kuenzi L, Chung S, Dennis JS, Baumlin N, Adewale AT, Rowe SM (2019). The effects of the anti-aging protein klotho on mucociliary clearance. Front Med (Lausanne).

[35] Papakonstantinou E, Roth M, Klagas I, Karakiulakis G, Tamm M, Stolz D (2015). COPD exacerbations are associated with proinflammatory degradation of hyaluronic acid. Chest.

[36] Galdi F, Pedone C, McGee CA, George M, Rice AB, Hussain SS, Vijaykumar K, Boitet ER, Tearney GJ, McGrath JA (2021). Inhaled high molecular weight hyaluronan ameliorates respiratory failure in acute COPD exacerbation: a pilot study. Respir Res.

[37] Rovina N, Dima E, Gerassimou C, Kollintza A, Gratziou C, Roussos C (2009). Interleukin-18 in induced sputum: association with lung function in chronic obstructive pulmonary disease. Respir Med.

[38] Nakajima T, Owen CA (2012). Interleukin-18: the master regulator driving destructive and remodeling processes in the lungs of patients with chronic obstructive pulmonary disease?. Am J Respir Crit Care Med.

[39] Krick S, Grabner A, Baumlin N, Yanucil C, Helton S, Grosche A, Sailland J, Geraghty P, Viera L, Russell DW (2018). Fibroblast growth factor 23 and Klotho contribute to airway inflammation. Eur Respir J.

[40] Gao W, Yuan C, Zhang J, Li L, Yu L, Wiegman CH, Barnes PJ, Adcock IM, Huang M, Yao X (2015). Klotho expression is reduced in COPD airway epithelial cells: effects on inflammation and oxidant injury. Clin Sci (Lond).

[41] Verde Z, Gonzalez-Moro JM, Chicharro LM, Reinoso-Barbero L, Bandres F, Gomez-Gallego F, Santiago C (2017). A paradox: alpha-Klotho levels and smoking intensity. Lung.

[42] Nakanishi K, Nishida M, Harada M, Ohama T, Kawada N, Murakami M, Moriyama T, Yamauchi-Takihara K (2015). Klotho-related molecules upregulated by smoking habit in apparently healthy men: a cross-sectional study. Sci Rep.

[43] Singh A, Ling G, Suhasini AN, Zhang P, Yamamoto M, Navas-Acien A, Cosgrove G, Tuder RM, Kensler TW, Watson WH, Biswal S (2009). Nrf2-dependent sulfiredoxin-1 expression protects against cigarette smoke-induced oxidative stress in lungs. Free Radic Biol Med.

[44] Wang L, Chen Q, Yu Q, Xiao J, Zhao H (2021). TREM-1 aggravates chronic obstructive pulmonary disease development via activation NLRP3 inflammasome-mediated pyroptosis. Inflamm Res.

[45] Churg A, Zhou S, Wright JL (2012). Matrix metalloproteinases in COPD. Eur Respir J.

[46] Kodal JB, Kobylecki CJ, Vedel-Krogh S, Nordestgaard Børge G, Bojesen SE (2018). *AHRR* hypomethylation, lung function, lung function decline and respiratory symptoms. Eur Respir J.

[47] Wu K, Byers DE, Jin X, Agapov E, Alexander-Brett J, Patel AC, Cella M, Gilfilan S, Colonna M, Kober DL (2015). TREM-2 promotes macrophage survival and lung disease after respiratory viral infection. J Exp Med.

[48] Perotin J-M, Polette M, Deslée G, Dormoy V (2021). CiliOPD: a ciliopathy-associated COPD endotype. Respir Res.

[49] Lesser M, Padilla ML, Cardozo C (1992). Induction of emphysema in hamsters by intratracheal instillation of cathepsin B. Am Rev Respir Dis.

[50] Kodal JB, Kobylecki CJ, Vedel-Krogh S, Nordestgaard BG, Bojesen SE (2018). AHRR hypomethylation, lung function, lung function decline and respiratory symptoms. Eur Respir J.

[51] Klagas I, Goulet S, Karakiulakis G, Zhong J, Baraket M, Black JL, Papakonstantinou E, Roth M (2009). Decreased hyaluronan in airway smooth muscle cells from patients with asthma and COPD. Eur Respir J.

[52] Hussain SS, George S, Singh S, Jayant R, Hu CA, Sopori M, Chand HS (2018). A small molecule BH3-mimetic Suppresses cigarette smoke-induced mucous expression in airway epithelial cells. Sci Rep.

[53] Kaza N, Lin VY, Stanford D, Hussain SS, Libby EF, Kim H, Borgonovi M, Conrath K, Mutyam V, Byzek SA (2021). Evaluation of a novel CFTR potentiator in copd ferrets with acquired cftr dysfunction. Eur Respir J.

