# Supplementary figures and images for: Comparative transcriptomics in human COPD reveals dysregulated genes uniquely expressed in ferrets
Source: Respir Res. 2022 Oct 10;23:277. doi: 10.1186/s12931-022-02198-0 (PMC9552453; doi:10.1186/s12931-022-02198-0)

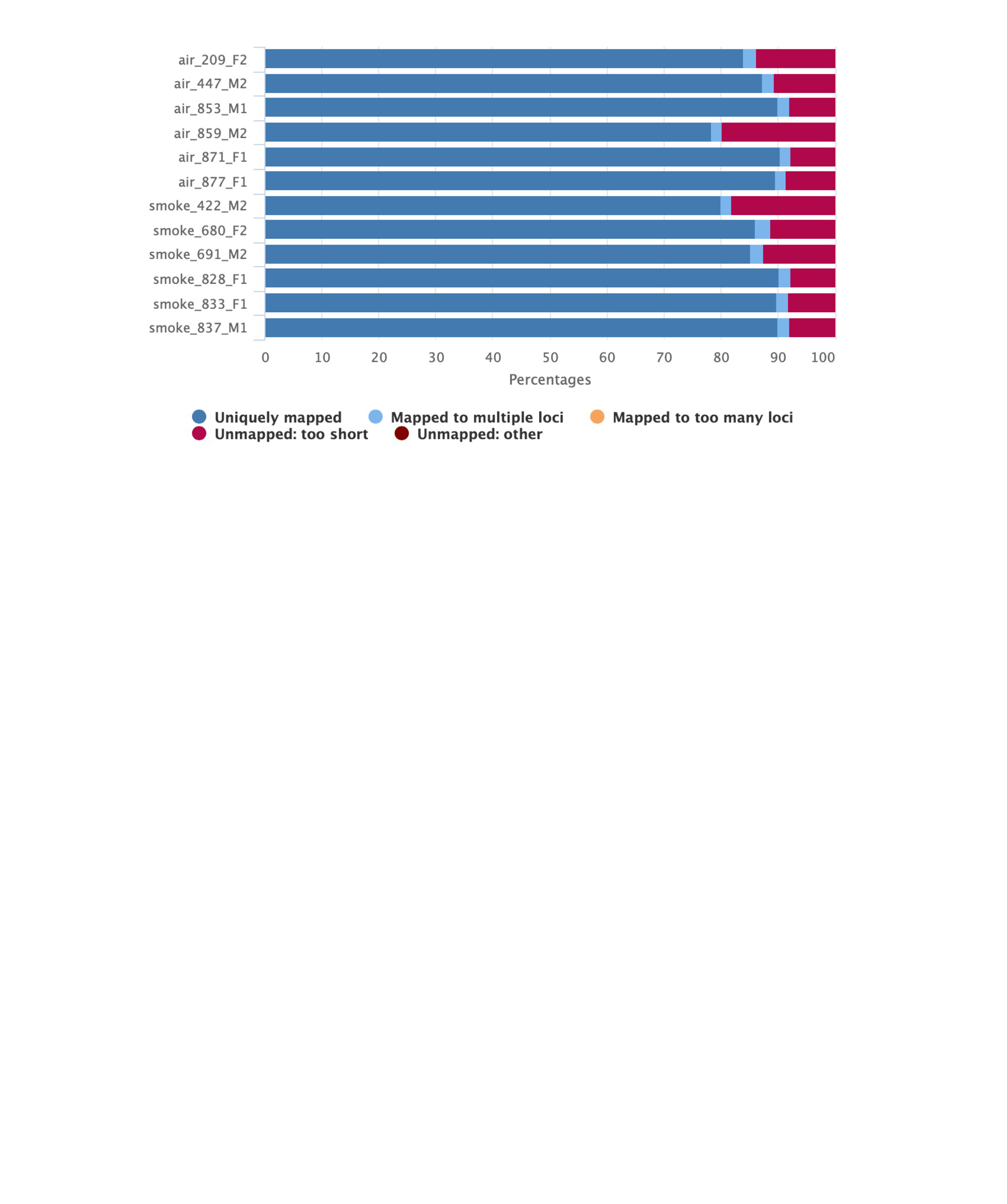

Supplement: Supplementary file 2 — Additional file 2: Fig. S1. The proportion of paired ends reads for the ferret lung samples, by individual ferrets. [file 12931_2022_2198_MOESM2_ESM.tif]
