# Supplementary material for: Comparative transcriptomics in human COPD reveals dysregulated genes uniquely expressed in ferrets
Source: Respir Res. 2022 Oct 10;23:277. doi: 10.1186/s12931-022-02198-0 (PMC9552453; doi:10.1186/s12931-022-02198-0)
Supplement: Supplementary file 3 — Additional file 3: Table S2. The number of paired ends reads for the ferret lung samples. [file 12931_2022_2198_MOESM3_ESM.docx]

**Supplemental Table 2.** The number of paired ends reads for the ferret lung samples.

| Category | Uniquely mapped | Mapped to multiple loci | Mapped to too many loci | Unmapped: too short | Unmapped: other | Total |
| --- | --- | --- | --- | --- | --- | --- |
| Air Control 1 | 36,696,922 | 802,698 | 8,971 | 3,226,998 | 16,298 | 40,751,887 |
| Air Control 2 | 51,613,865 | 1,125,045 | 9,139 | 4,353,208 | 17,139 | 57,118,396 |
| Air Control 3 | 43,223,685 | 943,937 | 8,908 | 4,080,477 | 14,487 | 48,271,494 |
| Air Control 4 | 33,526,441 | 927,466 | 3,673 | 5,457,059 | 7,984 | 39,922,623 |
| Air Control 5 | 36,568,140 | 899,794 | 4,300 | 4,441,631 | 4,194 | 41,918,059 |
| Air Control 6 | 42,674,956 | 1,101,285 | 5,437 | 10,718,445 | 5,452 | 54,505,575 |
| Smoke 1 | 33,648,837 | 847,894 | 3,577 | 7,579,385 | 4,208 | 42,083,901 |
| Smoke 2 | 30,982,289 | 950,498 | 3,685 | 4,016,176 | 3,599 | 35,956,247 |
| Smoke 3 | 31,015,814 | 811,047 | 3,530 | 4,558,295 | 3,641 | 36,392,327 |
| Smoke 4 | 33,648,837 | 847,894 | 3,577 | 7,579,385 | 4,208 | 42,083,901 |
| Smoke 5 | 30,982,289 | 950,498 | 3,685 | 4,016,176 | 3,599 | 35,956,247 |
| Smoke 6 | 31,015,814 | 811,047 | 3,530 | 4,558,295 | 3,641 | 36,392,327 |
